# The size dependency of foraging behaviour: an empirical test performed on aquatic amphipods

**DOI:** 10.1007/s00442-022-05195-8

**Published:** 2022-06-09

**Authors:** Francesco Cozzoli, Milad Shokri, Sarah Boulamail, Vanessa Marrocco, Fabio Vignes, Alberto Basset

**Affiliations:** 1 Research Institute on Terrestrial Ecosystems (IRET–URT Lecce), National Research Council of Italy (CNR), Campus Ecotekne, S.P. Lecce-Monteroni, 73100 Lecce, Italy; 2 Laboratory of Ecology, Department of Biological and Environmental Sciences and Technologies, University of the Salento, S.P. Lecce-Monteroni, 73100 Lecce, Italy

**Keywords:** Body size, Foraging behaviour, Patch use, Aquatic amphipods, Microcosm

## Abstract

The behavioural choices made by foragers regarding the use of resource patches have a direct influence on the energy balance of the individual. Given that several individual traits linked to the acquisition of spatially distributed resources increase with body size (e.g., energy requirements, resource ingestion rates, and movement capacity), it is reasonable to expect size dependencies in overall foraging behaviour. In this study, we tested how body size influences the number, duration, and frequency of foraging episodes in heterogeneous resource patches. To this end, we performed microcosm experiments using the aquatic amphipod *Gammarus insensibilis* as a model organism*.* An experimental maze was used to simulate a habitat characterised by resource-rich, resource-poor, and empty patches under controlled conditions. The patch use behaviour of 40 differently sized specimens foraging alone in the experimental maze was monitored via an advanced camera setup. Overall, we observed that individual body size exerted a major influence on the use of resource patches over time. Larger individuals had stronger preference for the resource-rich patches initially and visited them more frequently than smaller individuals, but for shorter periods of time. However, larger individuals subsequently decreased their use of resource-rich patches in favour of resource-poor patches, while smaller individuals continued to prefer resource-rich patches for the whole experimental time. With body size being a key organismal trait, our observations support the general understanding of foraging behaviours related to preference, patch use, and abandonment.

**Supplementary Information:**

The online version contains supplementary material available at 10.1007/s00442-022-05195-8.

## Introduction

Foraging decisions regarding the selection and abandonment of resource patches are crucial to individual fitness of motile foragers (MacArthur and Pianka 1966; Brown et al. 1988). For instance, optimal foragers should exploit a patch until the ingestion rate of the resources is higher than the average in the foraging area (MacArthur and Pianka 1966; Charnov 1976) or if/until the ingestion rate of resources balances the energy and fitness costs of foraging (Brown et al. 1988). Identifying the drivers of patch-scale foraging decisions is crucial to understanding population dynamics and large-scale patterns of space use (Potts et al. 2014; Van Moorter et al. 2016; Milles et al. 2020; Webber et al. 2020). The importance of body size as a driver of patch-scale foraging decision has long been recognized in ecological theory (Mittelbach 1981). However, the underlying mechanisms that determine the size dependence of foraging behaviour have not yet been fully investigated (Dial et al. 2008; Webber et al. 2020), hampering the mechanistic connection between size scaling and foraging theories (Hirt et al. 2018).

Larger body size implies higher energy costs (Kleiber 1932; West et al. 1997; Kooijman, 2000; Makarieva et al. 2004) which must be supported by higher ingestion of resources per unit of time (Peters 1983; Hendriks, 1999). It follows that larger foragers have greater ability to manage and ingest large amounts of resources (Vucic-Pestic et al. 2010), and, therefore, are faster in exploiting abundant resource patches (Basset et al. 2012). However, larger foragers are also limited in exploiting patches with (or until reaching) a low resource level, because these patches cannot satisfy their higher energy need (Kotler and Brown 1990; Basset 1995; Cozzoli et al. 2018; Cozzoli et al. 2020). For instance, larger rodents may achieve higher foraging rates due to size-related adaptations (Brown et al. 1994), but they abandon the patch at higher residual resource density (Cozzoli et al. 2019). Furthermore, larger foragers generally have higher dispersal ability (Innes and Houlihan 1985; Hirt et al. 2017) and lower locomotion costs per unit of body mass (Denny 1980; Dial et al. 2008), which make it more convenient for them to seek and move to new and more abundant resource patches than further exploit the patch in use.

**Fig. 1:**
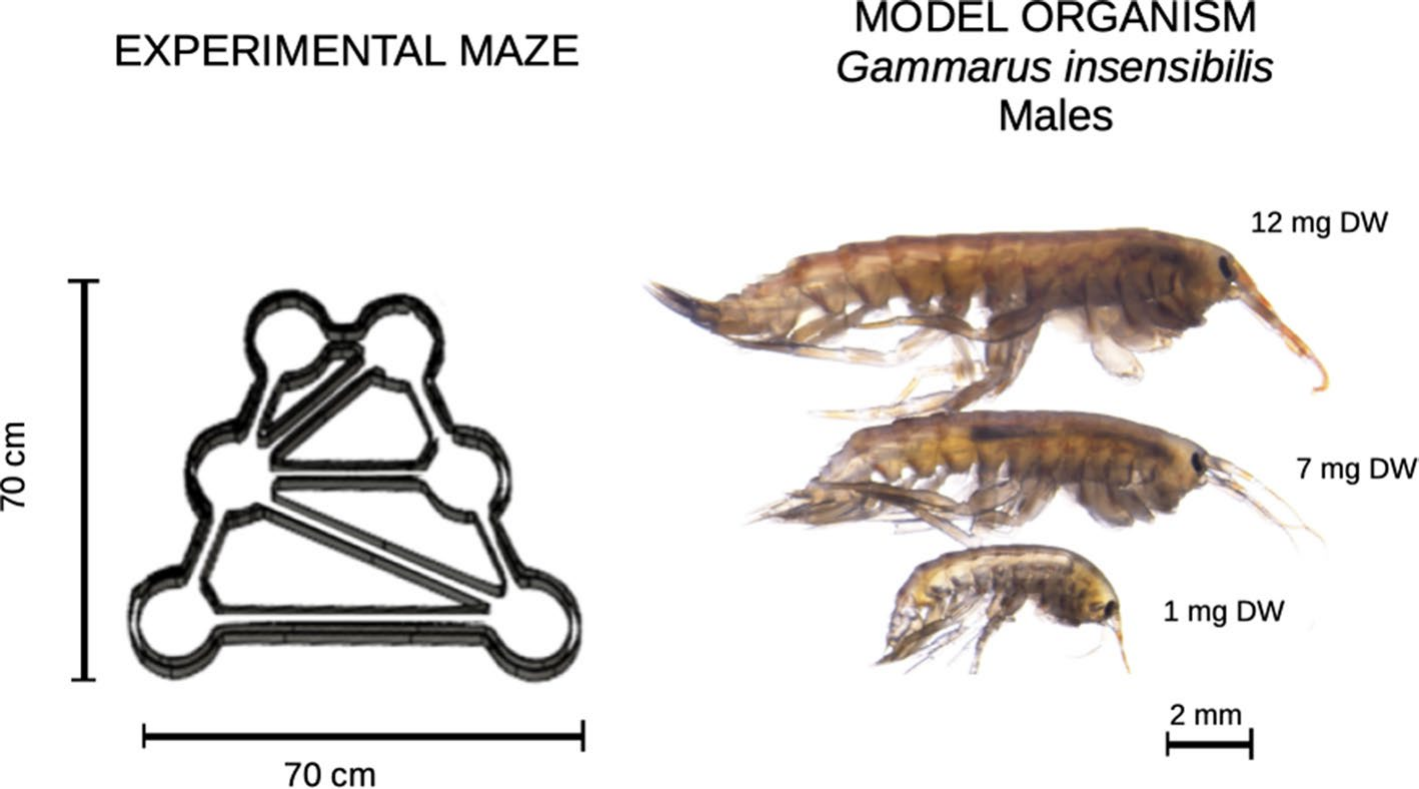
Left: sketch of the experimental maze. For each experiment trial, 1 g DW of leaves were placed in one of the maze’s patches and 0.5 g DW in another, leaving the other four empty. The selection of the resource patches was randomised to minimise the potential influence of microcosm geometry on the specimens’ behaviour. Right: as model organisms, we used differently sized male individuals of the aquatic amphipod *Gammarus insensibilis* (Stock, 1966)

Mobile foragers modulate their use of a resource patch mostly by adjusting the duration and the frequency of their foraging episodes (Van Moorter et al. 2016). Generally, the higher the preference for a patch, the more frequently foragers visit it and the more time they spend there. However, longer and more frequent foraging episodes may reduce the locally available amount of resources (Van Moorter et al. 2009). Since larger foragers are expected to: (i) ingest more resource per unit of time when resources are abundant; (ii) abandon resource patches at higher resource levels than smaller individuals due to their higher energy requirements; (iii) have higher dispersal ability, making them more inclined to move and explore the surrounding space; the choice, duration, and the frequency of visits to resource patches are expected to be size-dependent, as well. To test these hypotheses, we performed microcosm experiments using the aquatic amphipod *Gammarus insensibilis* (Stock, 1966) as a model organism. Thanks to a novel methodological approach (Shokri et al. 2021), we were able to accurately describe the patch use behaviour of *G. insensibilis* specimens in a range of sizes (Fig. 1). This allowed us to describe the observed patterns in individual foraging as allometric functions (see Glazier 2021) of the foragers’ body mass.

## Materials and methods

### Model organism

The biological model used, *Gammarus insensibilis* (Stock, 1966) (Fig. 1), is a widely distributed crustacean species found in European transitional and coastal waters, reaching a maximal length of 19 mm at adult size (Tillin and White 2017). They disperse over short distances by relatively fast swimming movements (up to 10 cm s^−1^) that are often interrupted by rest and feeding breaks of a few minutes (Shokri et al. 2021). *G. insensibilis* feed mainly by grazing microscopic fungi growing on submerged decaying plant litter (Nelson 2011). They are selective foragers, feeding preferentially on fungi with high lipid or protein content (Arsuffi and Suberkropp 1989). However, they exploit less palatable microorganisms or even the leaves matrix when their preferred food is in short supply (Rossi 1985; Graça et al. 1993; Aßmann et al. 2011; Mancinelli 2012). While feeding, *G. insensibilis* quickly scroll the surface of leaves for highly valuable resources (Basset 1992; Shokri et al. 2021).

### Experimental maze

The experimental maze was made of transparent Plexiglas^®^ and was composed of six circular patches (13 cm in diameter, 3 cm high) connected by a network of channels 2.5 cm wide and 3 cm high, of length variable between 5 and 42 cm (Fig. 1). A set of infrared-sensitive cameras (Basler aca1300–60gm) mounted above the maze was used to record the movement of individual specimens. An infrared light source was placed under the maze to have high contrast and facilitate specimen detection. The video-tracking files were processed automatically by Ethovision XT 14 software to determine which patches were visited, for how long and when. The frame supporting the cameras, the backlight unit, and the analytical software were developed by Noldus Information Technology BV. The mazes were kept in an isolated and temperature-controlled room (18 ± 0.3 °C) to prevent external disturbance that could interfere with the behaviour of the specimens. See Shokri et al. (2021) for further details.

### Trophic resource

As a trophic resource, we used leaves of *Phragmites australis*. The leaves were collected in early spring (at the same collection time as the animal specimens) in a single episode from a single location in the coastal area of the Acquatina lagoon (SE Italy, 40°26′31"N 18°14′19"E). Authorization for collection of the plant material was issued by the competent authority (the University of the Salento). In the laboratory, the leaves were cut into approximately 10 cm lengths, dried in the oven at 60 °C for 72 h, and weighed. The leaves were inoculated with water from the specimens’ collection site and conditioned for 2 weeks in circulating water at 18 °C before the experiment. The nutritional quality of the leaves is known to increase during conditioning because of microbial colonization and the assimilation of nutrients from the leaves by fungi and bacteria (Marks 2019).

### Experimental procedures

#### Specimens’ collection and keeping

The specimens of *G. insensibilis* used in this experiment were collected in the Le Cesine National Nature Reserve (Vernole, SE Italy, 40°21′38"N 18°19′49"E) 2 weeks before the experiments. They were transferred to the Biodiversity and Ecosystem Functioning Laboratory (*BIOforIU*) of the University of the Salento in thermo-insulated containers filled with water from the sampling sites and aerated during transport. Authorization for the collection of specimens was issued by the competent authority (World Wildlife Fund Italy). Specimens were maintained in the laboratory’s aquaria for 2 weeks at a temperature of 18 °C and salinity of 7 (i.e., similar to the conditions at the sampling site of the specimens) and fed with decaying *P. australis* leaves. Given that *P. australis* is by far the largest contributor to plant organic detritus at the *G. insensibilis* collection site (Tomaselli et al. 2011), we are confident that the specimens were accustomed to this type of resource. Only males were selected for laboratory experiments, since oocyte production in females may induce non-size-related variability in energy requirements (Glazier et al. 2011; Becker et al. 2016).

#### Resources patches’ preparation

For each experiment trial, 1 g dry weight (DW) of conditioned leaves was placed in one of the maze’s patches (thereby creating a resource-rich patch) and 0.5 g DW was placed in another (thereby creating a resource-poor patch), leaving the other four empty, thereby creating a heterogeneous environment. The positioning of the resource patches was randomised in each trial to minimize the potential influence of maze geometry on the specimens’ behaviour. We expected that during the experimental time (360 min), the specimens would selectively feed on the fungal biomass growing on the surface of the leaves rather than on the vegetal matrix (Graça et al. 1993; Mancinelli 2012). Therefore, they would explore the maze extensively even if they could theoretically support themselves on the resources present in a single patch (Basset 1992). As instantaneous growth rate of fungal biomass on submerged *P. australis* detritus is relatively low (ca. 0.7% d^−1^) and stable (Kominkova et al. 2000), we assumed that the growth of decomposing microorganisms after grazing was either negligible in the experimental timeframe (6 h) or constant across patches. The specific amount of resources used in this experiment to simulate resource-rich and resource-poor patches was determined through preliminary observation of our experimental systems. Lower amounts of resource than used did not allow to clearly distinguish differences in foraging. Greater amounts of resources had the effect of increasing the specimens’ residence time on the patches, making the overall experiment duration impractical.

#### Experimental trials

Prior to the experiment, specimens of different size were selected on the base of their body length, starved for 24 h to ensure similar starting conditions in individual resource requirements (Shokri et al. 2021). Using a constant starvation time for differently sized individuals might generate uncertainty in our observations due to size dependency in starvation resistance (Gergs and Jager 2014). However, lacking detailed a priori knowledge on the size-dependent behavioural response of *G. insensibilis* to starvation, we chose a constant time of 24 h, because it generally ensures physiological and metabolic equilibrium among differently sized individuals of aquatic invertebrates (Glazier et al. 2011; Shokri et al. 2019).

Each specimen was tested alone without replication, *i.e.,* 40 separate experiments were conducted, each with a single specimen in the maze. Each trial lasted 6 h and it was carried out at the same time of day (9.00 to 15.00) at controlled temperature (18 °C). The resource patches were placed in the maze 30 min before introducing the specimen, which were always released in one of the empty patches. During the experiment, we considered a patch “visited” if the tested specimen travelled the whole length of a channel from a neighbouring patch, entered the patch with its whole body, and remained there for at least 30 s. Thus, brief "probes" of patches lasting less than 30 s (which were also sporadically observed) were not considered to be foraging episodes for the purposes of the analysis. During these “probes”, the specimens were probably still able to evaluate the amount of resource present in the patch as they briefly explored the leaves' surface. After the experiment, the specimens were dried individually in an oven at 60 °C for 72 h and weighed to the nearest ± 0.001 mg. The experiments were conducted in two separate time blocks (spring 2020 and autumn 2021), each block comprising 20 specimens.

### Data analysis

The number of visits to type of patches (N) and number of visits to the resource patches only (N) during the whole experiment were used as a proxy for the individual cumulative space use and modelled as a function of the foragers individual body mass (mg DW). Since the relationships between body size and amount of space used are commonly formulated as a power law (Tamburello et al. 2015; Glazier 2021), the response and the explanatory variable were log-transformed. The two time blocks of measurements were fitted as random terms.

To investigate the change in the behaviour of foragers across time, the duration of the visits (h) to the type of patch defined according to the amount of resource originally present (empty, resource-poor or resource-rich) was modelled as a function of the fixed terms individual body mass (mg DW), starting time of the visit (h) and patch(es) type. Similarly, the interval between consecutive visits (h) to the same type of patch (empty, resource-poor or resource-rich) was modelled as a function of the fixed terms individual body mass (mg DW), ending time of the visit (h) and patch(es) type. Two-way interactions were included as fixed terms in both models. To account (i) for variations across the time blocks of measurements and (ii) for the non-independence of observations repeated over time on the same individual, we allowed random variation in intercept at the level of individuals nested within time blocks. The response variables’ duration of visits and interval between visits, and the explanatory variable body mass of the foragers were log-transformed to model the size dependency as a power law.

We quantified overall patch preferences of foragers by dividing the experimental time of 360 min into 72 slots of 5 min each. Each specimens’ foraging behaviour was expressed in terms of type of patch mainly frequented during each time slot. Pooling together observations from all specimens, this approach allows to quantify the overall patch preferences of the foragers, i.e., the fraction of individuals using a certain patch type at a given time during the experiment. Logistic regression was used to model variation in preferred patches with respect to the following fixed terms: experimental time (h), type of used patch (empty, resource-poor or resource-rich), and forager body mass (mg DW). Also in this case, the model was fitted with two-way interactions between fixed terms; the individuals nested within experimental blocks were used as random term; the explanatory variable body mass was log-transformed.

Uncertainty of model estimates was reported as 95% Confidence Interval [lower–upper]. All analyses were performed within the R free software environment (R Core Team 2019) using the lme4 (Bates et al. 2015) and sjPlot (Lüdecke 2018) packages. The full dataset is available as an appendix to this paper (Appendix A) and in the OSF repository (https://doi.org/10.17605/OSF.IO/GHVKN).

## Results

### Preliminary observations

The specimens used in this experiment (*N* = 40) had body masses ranging from 0.6 mg DW to 12.4 mg DW (average value 6.2 mg DW [± 4.1 SD]) (Fig. 1). During the experiment, the specimens moved continuously around the maze, alternating periods of exploitation of the resource patches with rapid exploration of the surrounding environment. Although the specimens also carried out activities other than foraging, while in the resource patches (e.g., hiding, resting, and intra-patch movements), much of their time was allocated to feeding. The number and/or duration of foraging episodes can thus be considered a positive measure of the resource patch value perceived by the foragers.

### Fixed vs random effects

The mixed regression models were able to explain from 22% (interval between visits) to 50% (total number of visited patches) of the observed variance of patch usage behaviour descriptors on the base of the fixed terms. The estimated random variation across experimental blocks (*N* = 2) and individuals (*N* = 40) was virtually null (Tables 1–2), implying that our observations are highly replicable and that the behavioural pattern followed by different individuals is constant under the same external conditions.

**Table 1:** Summary of the mixed regression model of the total number of visits to patch types during the complete experimental time (*N*, left) and the number of the visits to the resource patches only (*N*, right) with respect to the foragers’ individual body mass (*M*, mg DW), accounting for random variation in intercept at the level of experimental blocks

	log(N of visits to all patches)			log(N of visits to resource patches only)		
Predictors	Estimates	95% CI	*p*	Estimates	95% CI	*p*
(Intercept)	2.47	2.23 to 2.71	< 0.001	2.06	1.79 to 2.32	< 0.001
log(*M*)	0.46	0.31 to 0.60	< 0.001	0.35	0.19 to 0.51	< 0.001
Random effects						
* σ*^2^	0.18			0.22		
* τ*_00_	< 0.01 _Block_			< 0.01 _Block_		
* N*	2 _Block_			2 _Block_		
Observations	40			40		
Marginal *R*^2^/ conditional *R*^2^	0.50/0.50			0.33/0.33

**Table 2:** Summary of the mixed regression models of duration of visits to patches (h, left), time to return to the patches (h, centre) and individuals preference for patch typologies (%, right) with respect to the fixed terms experimental time (h), amount of resource originally present in the patch (Rich, Poor or Empty) and forager body mass (*M*, mg DW), accounting for random variation in intercept at the level of individuals nested within experimental blocks

Predictors	log(Duration of visit)			log(Interval between consecutive visits)			logit(Preference)		
	Estimates	95% CI	*p*	Estimates	95% CI	*p*	Estimates	95% CI	*p*
Intercept	– 2.21	– 2.62 to – 1.81	< 0.001	– 0.27	– 0.78 to 0.25	0.310	0.09	0.06 to 0.15	< 0.001
Rich	2.05	1.63 to 2.46	< 0.001	– 3.37	– 3.95 to – 2.79	< 0.001	145.70	89.19 to 238.02	< 0.001
Poor	0.61	0.08 to 1.15	0.024	0.33	– 0.39 to 1.06	0.370	0.50	0.30 to 0.86	0.012
Time	– 0.12	– 0.23 to – 0.01	0.034	– 0.25	– 0.39 to – 0.10	< 0.001	1.25	1.12 to 1.38	< 0.001
log(*M*)	– 0.36	– 0.57 to – 0.15	< 0.001	– 0.43	– 0.69 to – 0.18	< 0.001	1.75	1.42 to 2.17	< 0.001
Rich × Time	– 0.29	– 0.38 to – 0.20	< 0.001	0.56	0.44 to 0.69	< 0.001	0.52	0.47 to 0.58	< 0.001
Poor × Time	– 0.06	– 0.17 to 0.04	0.249	– 0.17	– 0.32 to – 0.03	0.016	1.20	1.08 to 1.33	< 0.001
Rich × log(*M*)	– 0.19	– 0.37 to – 0.02	0.032	0.77	0.54 to 1.00	< 0.001	0.33	0.27 to 0.40	< 0.001
Poor × log(*M*)	– 0.06	– 0.27 to 0.15	0.591	0.44	0.16 to 0.72	0.002	0.79	0.65 to 0.97	0.024
Time × log(*M*)	0.08	0.03 to 0.13	0.002	– 0.03	– 0.10 to 0.04	0.401	1.00	0.95 to 1.05	0.927
Random effects									
* σ*^2^	0.84			1.49			3.29		
* τ*_00_	0.08 _Individual:Block_			< 0.01 _Individual:Block_			< 0.01 _Individual:Block_		
	< 0.01 _Block_			< 0.01 _Block_			< 0.01 _Block_		
* N*	40 _Individual_			40 _Individual_			40 _Individual_		
	2 _Block_			2 _Block_			2 _Block_		
Observations	908			908			4320		
Marginal *R*^2^/conditional *R*^2^	0.28/0.28			0.22/0.22			0.27/0.27		

### Total number of visited patches

**Fig. 2:**
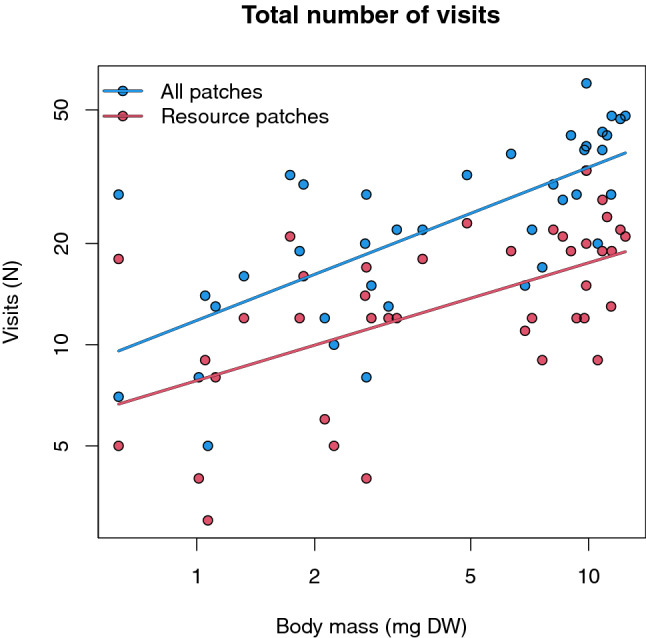
Total number of visits to patch types during the experiment (*N*) for the whole maze (blue) and number of visits to the resource patches only (red). Regression models are summarised in Table 2

The total number of (re)visits to patches type by each forager ranged from 5 to 60, while the number of visits to the resource patches ranged from 3 to 33. Both the total number of (re)visited patch types and the number of visits to resource patches significantly (*p* < 0.001) increased as a hypoallometry of the foragers’ body mass (Fig. 2, Table 1).

### Duration of visits to the same patch type

**Fig. 3:**
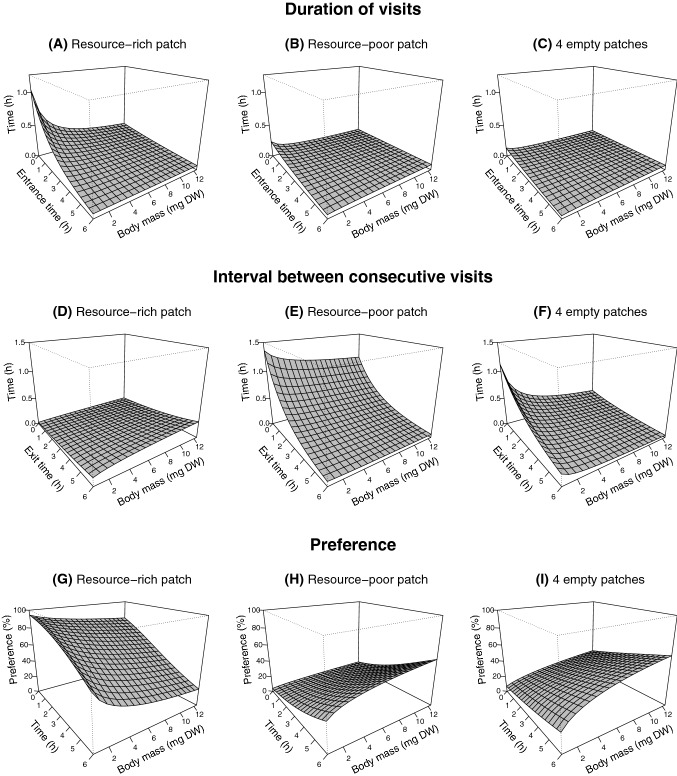
Model response surfaces of duration of visits to patches (h) (**A**–**C**), interval between consecutive visits (h) (**D**–**F**), and preference for patch typologies (%) (**G**–**I**) with respect to the fixed terms experimental time (h) and forager body mass (mg DW). Regression models are summarised in Table 1

At the beginning of the experiment, the foragers made significantly (*p* < 0.001) longer visits to the resource-rich patch (estimated 17 min [14–21] for average-sized individuals) than to the resource-poor patch (estimated 5 min [4–8] for average-sized individuals) and to the empty patches (estimated 3 min [3–4] for average-sized individuals) (Fig. 3A–C, Table 2). The duration of the visits to the same patch type decreased significantly (*p* = 0.034) over time, in particular for visits to the resource-rich patch (*p* < 0.001) (Fig. 3A–C, Table 2). The duration of visits to the same patch type also decreased significantly (*p* < 0.001) at the increase of the body mass of the foragers, in particular for visits to the resource-rich patch (*p* = 0.032) (Fig. 3A–C, Table 2). However, the decrease over time in duration of visits was slower at the increase of the foragers’ body mass (*p* < 0.01). At the end of the experiment, the estimated duration of visits was of ca. 4 min independent of the type of patch and of the body mass of the forager (Fig. 3A–C, Table 2).

### Interval between consecutive visits to the same patch typology

At the beginning of the experiment, the foragers abandoned the resource-rich patch for short intervals before returning to it (estimated 3 min [2–4] for average-sized individuals). The initial estimated time interval between consecutive visits of average-sized foragers to the resource-poor patch and to the empty patches, respectively, was of 63 min [39–101] and 21 min [16–28] (Fig. 3D–F, Table 2). Over time, the time interval between consecutive visits to the resource-rich patch increased significantly (*p* < 0.001). Simultaneously, the time interval between consecutive visits to the resource-poor patch and to the empty patches decreased (*p* < 0.001) (Fig. 3D–F, Table 2). The interval between visits to the resource-poor patch and to the empty patches decreased significantly (*p* < 0.001) at the increase of the forager's body mass (Fig. 3D–F, Table 2). Conversely, the time intervals between visits to the resource-rich patch increased with the forager's body mass (*p* < 0.001) (Fig. 3F, Table 2).

### Preference for patch type

At the beginning of the experiment, the near totality of foragers preferred the resource-rich patch (Fig. 3G, Table 2). Over time, the foragers’ preference decreased significantly (*p* < 0.001) for the resource-rich patch (Fig. 3G, Table 2) and, simultaneously, increased significantly their preference (*p* < 0.001) for the resource-poor patch and for the empty patches (Fig. 3H–I, Table 2). As the foragers’ body mass increased, the preference for both resource patches decreased (*p* < 0.001) and preference for empty patches increased (*p* < 0.001) (Fig. 3G-I, Table 2).

## Discussion

Overall, we detected a clear influence of specimens’ body mass on the use of resource patches. We also observed that the amount of resource present in a patch has a mass-dependent influence on the selection and abandonment of the resource patch.

Independent of body mass, the foragers exploited both the resource-rich and the resource-poor patches in repeated visits interspersed with moments of exploration of the surrounding environment and exploitation of the other resource patch. The vast majority of the long-lasting visits to the resource patches occurred in the early hours of the experiment, when, we assume, the previously starved foragers consumed most of the resource. Subsequent visits were progressively shorter in duration, suggesting that only part of the available resource was consumed during each foraging episode. This pattern could be explained by the fact that *G. insensibilis* are selective feeders, exploiting at first the most palatable part of the resource (Rossi 1985; Aßmann et al. 2011; Mancinelli 2012) and preferentially moving in search of other valuable resources rather than making full use of the patch under exploitation. This also implies that perceived patch value decreases with each foraging episode at a higher rate than can be inferred from the amount of residual resource alone, since a decrease in quality also occurs (Price and Correll 2001). This patch use strategy may have been selected, because it also reduces predation risk (Eccard et al. 2020) and compensates for incomplete knowledge of patch quality by migrating between patches repeatedly and thus mitigating incorrect patch choices (Cressman and Krivan 2006).

Initially, the larger foragers mostly ignored the resource-poor patch, visiting it for a very short time and not returning. The smaller foragers, while preferring the rich resource patch, sometimes used the poor resource patch at the beginning of the experiment. This indicates that, when the resources were still abundant, smaller foragers do not strictly differentiate between the two resource patches as they are able to satisfy their low energy requirements in both cases (Cozzoli et al. 2020). Larger foragers were, in contrast, likely to achieve optimal ingestion rates in the resource-rich patch and therefore selected it first (Holling 1992; Basset 1995).

As experimental time progressed, the larger foragers visited the resource-poor patch or empty patches more frequently and for a longer time. In contrast, the smaller foragers continued the frequentation of the resource-rich patch until the end of the experiment. Our interpretation is that larger foragers, having higher ingestion rates (Peters 1983; Hendriks 1999), took proportionally less time to lower the resource in the resource-rich patch to a level at which they preferred to allocate time to the resource-poor patch or the exploration of the empty patches. Moreover, larger foragers should be unable to exploit low density resources (Basset et al. 2012; Cozzoli et al. 2018), which should further shorten the residence time in the initially exploited resource-rich patch. Conversely, the smaller foragers probably did not fully deplete the resource-rich patch and continued to exploit it for the duration of the experiment.

Although constrained in the same space and with an identical distribution of resources, the larger foragers visited a larger number of patches and therefore covered a cumulatively greater portion of space during the experimental time. In addition to faster resource exploitation and higher giving-up densities, the higher speed of larger foragers (Innes and Houlihan 1985; Hirt et al. 2017) as well as the lower locomotion costs per unit mass and distance travelled (Denny 1980; Spaargaren 1999) may contribute to their greater propensity to explore the surrounding environment instead of remaining in a resource patch.

Size-dependent variation in individual energy requirements has been shown to affect several components of foraging behaviour, such as patch selection (Auer et al. 2020), foraging effort (Biro et al. 2018; Cornwell et al. 2020), and patch abandonment (Cozzoli et al. 2018, 2020). Therefore, the positive size scaling of individual metabolic rates with body mass could offer a mechanistic interpretation to the pattern observed in our experiments: larger individuals explore more space and exploit resources at a faster rate to fulfil their higher energy requirement (Careau et al. 2008). It follows that some of the unexplained variance we observed may result from non-size-related variation in energy requirements (Shokri et al. 2019). It should also be considered that there are interdependencies between individual’s energy requirements and behaviour (Glazier 2015), particularly active behaviours being energetically expensive and resulting in greater resource requirements (Halsey et al. 2015). It is therefore possible that individuals who spent more time on resource patches reduced their energy requirements. Furthermore, small individuals may adopt strategies aimed at reducing activity and diverting energy towards growth (Killen et al. 2007) or may differ in anti-predator behaviour (Gavini et al. 2020), which could also explain the lower frequency and longer stays in the resource patches that we observed with increasing size. New experiments involving accurate measurements of the individual forager's metabolic rate are needed to better understand the relationship between body sizes, energy requirements, and patch exploitation behaviour. In addition, the role of individual personality (i.e., consistent difference between individuals in their behaviour across time and context, Dingemanse et al. 2010) should be investigated, because it could modulate the effect of size on resource exploitation (Cash-Padgett and Hayden, 2020; Cornwell et al. 2020) and foragers’ movement patterns (DiNuzzo and Griffen 2020; Milles et al. 2020).

### Implications

The criteria used by foragers of varying size for resource patch selection and abandonment may provide a link between individual energy requirements and spatial and temporal variation in the resource landscape (Van Moorter et al. 2016; Auer et al. 2020). For instance, the extent to which animals are able to locally deplete resources should determine home range size (Mitchell and Powell 2004). Staying longer in one patch before moving to another (as observed with smaller foragers) should favour more systematic coverage and should therefore lead to relatively smaller home ranges (Kodric-Brown and Brown 1978; Paton and Carpenter 1984). In addition, having a higher capacity for movement and larger home ranges leads to fewer returns to individual patches (and longer periods between them), including those incompletely exploited during the first foraging episodes (Seidel and Boyce 2015). Therefore, the pattern observed in our experiment is consistent with the observation of steep positive scaling of home range with individual body size (Ofstad et al. 2016).

Although our experiments were carried out on a single species and on individuals foraging alone, the observed patterns in patch exploitation behaviour support the hypothesis of size-related coexistence mechanisms even with regard to a single resource (Wilson 1975; Basset 1995; Szabó and Meszéna 2006; Basset and DeAngelis 2007). Indeed, the inability of larger foragers to exploit patches down to a low level of resources may enable smaller foragers to satisfy their requirements with what remains, and therefore to establish home ranges nested within those of their larger competitors (Basset 1995; Basset and DeAngelis 2007).

Our study highlights the importance of body size on foraging behaviours related to preference, patch use, and abandonment. Given the allometric links between body size, metabolism, home range, population density at carrying capacity, and potential coexistence, our observations can be included in a broader conceptual framework and contribute to the general understanding of space and resources use patterns in ecological systems.

## Supplementary Information

Below is the link to the electronic supplementary material.

Supplementary file1 (CSV 13 KB)
